# Effects of pre- and postnatal deletion of the transcription factor NKx2-1 on the expression of NGF, trkA, trkB and p75NTR in mice

**DOI:** 10.1186/1753-6561-9-S1-A6

**Published:** 2015-01-14

**Authors:** S Ersözlü, L Magno, T Naumann

**Affiliations:** 1 Institute of Cell Biology and Neurobiology, Centre of Anatomy, Charité Universitätsmedizin Berlin, Charitéplatz 1, 10117 Berlin, Germany; 2 Wolfson Institute for Biomedical Research and Department of Cell and Developmental Biology, University College London, London, UK

## Background

Expression of the homeodomain-containing transcription factor Nkx2-1 is essential for the prenatal development of the forebrain, lungs and thyroid gland. Humans with only one functioning NKX2-1-allele, a rare genetic condition known as “NKX2-1 haploinsufficiency”, become phenotypic in early childhood. This syndrome displays a variable combination of symptoms including congenital hypothyroidism, respiratory distress syndrome and choreoathetosis. The severity of the neurological symptoms varies between the patients but, however, little is known about the cell-types, which might be affected by the mutation. Recently it was shown in mice that cholinergic and parvalbumin (PV)-containing GABAergic neurons of the subcortical basal ganglia and magnocellular nuclei of the ventral forebrain maintain Nkx2-1 synthesis throughout life. It is well-known that postnatal cholinergic basal forebrain neurons are strongly influenced by the synthesis of neurotrophins including the “nerve growth factor” (NGF). For instance, NGF is essential for the differentiation and maintenance of these cells which otherwise, in the absence of this trophic molecule, undergo shrinkage and degeneration. It is also known that cortical GABAergic neurons, which are mostly described as their target cells, synthesize this factor. In order to elucidate whether impaired synthesis of Nkx2-1 is accompanied by corresponding effects on the neurotrophin synthesis, we have investigated conditional knock-out mice with either pre- or postnatal deletion of Nkx2-1. Additionally, we investigated the effects of these two mutations on the synthesis of corresponding neurotrophin receptors (trkA, trkB, p75NTR).

## Methods

Expression levels of NGF, p75NTR, trkA, trkB and two house-keeping genes (HPRT; GAPDH) were measured using quantitative real-time-PCR (qRT-PCR) in 20 mice. Five mutants (GADcre/+;Nkx2-1c/c, “prenatal mutation” & ChATcre/+;Nkx2-1c/c, “postnatal mutation”) and five controls each (GAD+/+;Nkx2-1fl/fl & ChAT+/+;Nkx2-1fl/fl) were used. The present study was carried out according to the institutional guidelines for animal welfare.

## Results

Significantly decreased expression levels of trkA (-82%) and p75NTR (-60%) were detected in GAD67-mutants, and of trkA (-56%) in ChAT-mutants. No significant changes were observed for NGF and trkB in both mutant lines and for p75NTR in ChATcre/+;Nkx2-1c/c mice.

## Conclusions

Our results can be summarized as follows: (1) the reduction in the number of cholinergic neurons after pre- and postnatal Nkx2-1-deletion is indeed accompanied by decreased levels of neurotrophin receptors but (2) cortical GABAergic NGF-synthesis is not impaired by the mutations. This suggests that permanent synthesis of NGF in the target regions of cholinergic basal forebrain neurons does not compensate for the effects of Nkx2-1 targeting.

